# Predicting Response to Neoadjuvant Therapy in Oesophageal Adenocarcinoma

**DOI:** 10.3390/cancers14040996

**Published:** 2022-02-16

**Authors:** William Jiang, Jelske M. de Jong, Richard van Hillegersberg, Matthew Read

**Affiliations:** 1 Upper Gastrointestinal Surgery Department, St Vincent’s Hospital Melbourne, 41 Victoria Parade, Fitzroy, VIC 3065, Australia; 2 Gastrointestinal Oncology Department, The University Medical Centre Utrecht, Heidelberglaan 100, 3584 CX Utrecht, The Netherlands; j.m.dejong-27@umcutrecht.nl (J.M.d.J.); r.vanhillegersberg@umcutrecht.nl (R.v.H.)

**Keywords:** oesophageal adenocarcinoma, gastroesophageal adenocarcinoma, oesophageal cancer, predictive biomarker, imaging marker, predict response, neoadjuvant therapy, chemotherapy, radiotherapy, chemoradiotherapy

## Abstract

**Simple Summary:**

Oesophageal adenocarcinomas are a distinct subtype of oesophageal cancer that has an increasing incidence in western countries. As these cancers are often late presenting, patients with locally advanced oesophageal adenocarcinomas are routinely treated with neoadjuvant chemotherapy with or without radiotherapy prior to surgery. Unfortunately, this neoadjuvant protocol demonstrates limited response, while exposing patients to the side effects of therapy. Biomarkers that can accurately predict neoadjuvant therapy response would save time, suffering, hospital resources and potentially improve survival.

**Abstract:**

(1) Background: Oesophageal cancers are often late-presenting and have a poor 5-year survival rate. The standard treatment of oesophageal adenocarcinomas involves neoadjuvant chemotherapy with or without radiotherapy followed by surgery. However, less than one third of patients respond to neoadjuvant therapy, thereby unnecessarily exposing patients to toxicity and deconditioning. Hence, there is an urgent need for biomarkers to predict response to neoadjuvant therapy. This review explores the current biomarker landscape. (2) Methods: MEDLINE, EMBASE and ClinicalTrial databases were searched with key words relating to “predictive biomarker”, “neoadjuvant therapy” and “oesophageal adenocarcinoma” and screened as per the inclusion and exclusion criteria. All peer-reviewed full-text articles and conference abstracts were included. (3) Results: The search yielded 548 results of which 71 full-texts, conference abstracts and clinical trials were eligible for review. A total of 242 duplicates were removed, 191 articles were screened out, and 44 articles were excluded. (4) Discussion: Biomarkers were discussed in seven categories including imaging, epigenetic, genetic, protein, immunologic, blood and serum-based with remaining studies grouped in a miscellaneous category. (5) Conclusion: Although promising markers and novel methods have emerged, current biomarkers lack sufficient evidence to support clinical application. Novel approaches have been recommended to assess predictive potential more efficiently.

## 1. Introduction

Oesophageal cancer is the sixth most common cause of cancer-related death worldwide. It is an aggressive and often-late presenting cancer associated with a poor 5-year survival rate of 15–25% [1]. The two main histological subtypes of oesophageal cancer include oesophageal squamous cell carcinoma (OSCC) and oesophageal adenocarcinoma (OAC). Although commonly homogenised in studies, these entities are histologically, epidemiologically and genetically distinct [2]. Over the last four decades, the incidence of OAC has increased dramatically in developed nations in contrast to declining OSCC rates, likely reflecting a concurrent rise in OAC risk factors, such as obesity and GORD and a decline in OSCC risk factors, such as smoking [2]. GORD and obesity are also independent risk factors for the development of Barrett oesophagus, the metaplastic transformation of squamous oesophageal epithelium into columnar intestinal-type mucosa, a further risk factor for OAC. Population level studies demonstrate that increasing GORD symptom severity and duration is associated with increased OAC risk with odds ratios of 2.80 (95% CI: 1.60, 4.91) and 6.24 (95% CI: 3.37, 11.55) for less than 10 years and 20 years or more of exposure, respectively [3]. OAC symptom onset may include dysphagia, odynophagia, progressive weight loss and fatigue from occult blood loss; however, the insidious nature of OAC often results in late presentation once the tumour has already reached a locally advanced or metastatic stage.

Despite their similar anatomical location, OAC shares more genetic resemblance with gastric cancer than OSCC, mostly owing to the amount of gross chromosomal instabilities shared between gastric cancers and OAC [4]. OAC also possesses a relatively high point mutation burden and tumoural clonal heterogeneity, which has therapeutic implications, as OAC is more chemo- and radioresistant than OSCC [2]. Moreover, OAC’s clonal heterogeneity contributes greater variability in dysregulated genes compared to OSCC, making the search for targeted therapies in OAC more challenging [2]. Given the histological and genetic heterogeneity in oesophageal cancer, this review will focus on OAC.

The treatment of OAC largely depends on its stage, as determined by the American Joint Committee on the Cancer TNM staging system and the medical fitness of the patient. OACs that are confined to the mucosa in the absence of local invasion, lymph node involvement and metastases, can be excised endoscopically often in combination with radiofrequency ablation. Locally advanced OAC, however, is treated multimodally with the combination of neoadjuvant chemotherapy or chemoradiotherapy prior to surgical resection. The aim of this neoadjuvant or preoperative treatment is to reduce tumour burden, destage and eliminate micrometastases. Currently, there are multiple different neoadjuvant regimens being used around the world and debate exists regarding the optimal modality. The CROSS trial presented a neoadjuvant chemoradiotherapy regimen consisting of weekly carboplatin and paclitaxel with concurrent radiotherapy to extend median overall survival compared with patients who had surgery alone (43.2 (24.9–61.4) months in the neoadjuvant therapy group vs. 27.1 (13.0–41.2) months in the surgery only group) [5]. The MAGIC trial introduced a regimen of epirubicin, cisplatin and infused fluorouracil, which demonstrated 5-year survival rates of 36.3% (95% CI: 29.5–43.0%) in the chemotherapy group compared to 23% (95% CI: 16.6–29.4%) in the surgery only arm [6]. Regardless of regimen, undertaking neoadjuvant therapy (NAT) is not without risks, as almost one in four patients on NAT will experience toxicity-related morbidity, with mortality rates of 1–3% reported [7]. Around 10-16% of patients on NAT do not make it to surgical resection for reasons including irresectability, tumour progression, death during treatment or medical deconditioning [7]. Moreover, less than one third of patients with locally advanced OAC derive a clinically meaningful response to NAT [8]. Therefore, there is an urgent need to find and develop biomarkers to accurately predict response to NAT.

The successful development of accurate predictive biomarkers for NAT response would have significant clinical implications on OAC treatment guidelines, potentially allowing clinicians to divert known NAT non-responders to earlier surgical intervention. Response to NAT in a surgical resection specimen is routinely graded in accordance with a tumour regression grade (TRG) system, of which Mandard’s TRG is one of the more widely accepted [9]. As molecular pathways involved in NAT responsiveness are not currently well understood, most studies thus far have relied on various imaging and molecular techniques to detect divergences between tissue samples of responders and non-responders to elucidate potential biomarkers. The result is a heterogeneous landscape of biomarkers at varying stages of pre-clinical and clinical investigation.

The aim of this review will be to explore the landscape of imaging and molecular predictive biomarkers, highlight promising biomarkers, and provide general and specific critique of the included studies. Developing predictive biomarkers for NAT response will contribute to making personalized medicine a reality for patients with OAC and save time, suffering, hospital resources and potentially improve survival.

## 2. Materials and Methods

On 27 August 2020, MEDLINE (accessed with PubMed and OVID), EMBASE (accessed with OVID) and ClinicalTrials.gov databases were searched using the search strategy outlined in Table A8 without limits. This search was derived from a population, intervention, comparator, outcome (PICO) model (without comparator) and references lists were screened for studies not captured within the search strategy.

All peer-reviewed full-text articles and conference abstracts were included if greater than 50% of the patient sample had OAC that required neoadjuvant therapy and were published after 2010 (Table A9). The decision was made to include conference abstracts as many novel methods and biomarkers have emerged recently and have not yet been reported in full-text articles. Only current clinically accepted neoadjuvant therapy regimens were included [5,6,10,11] (Table A10), though variations within the same drug class were also accepted. A marker or biomarker to predict neoadjuvant therapy outcome needed to be investigated within the paper. Non-English articles as well as review articles were excluded. To minimise heterogeneity, articles with 50% or less OAC patients were excluded unless the biomarkers analysis was unpooled by tumour subtype.

## 3. Results

**Figure A1:**
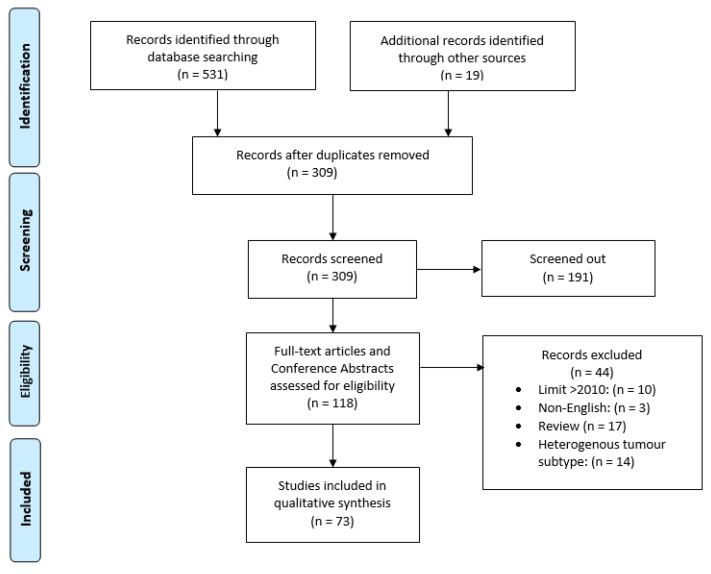
PRISMA flow diagram.

The initial search process yielded 550 results, which were screened by title, abstract and full text where necessary by the primary researcher (WJ). A total of 242 duplicates were removed, and 191 articles were screened out. In concordance with the exclusion criteria, 44 articles were excluded, leaving 73 full texts, conference abstracts and clinical trials eligible for review (Figure A1).

Included studies were compared based on: year published, sample size and tumour type, NAT regimen, TRG, study design and findings. Biomarkers were grouped into seven major categories based on their nature. These included: imaging, epigenetic, genetic, protein, immunologic, blood and serum-based markers. The remainder of studies were included under a miscellaneous category.

Multiple TRG systems were used between studies and the main ones have been summarised in Table A11 [9,12,13,14,15]. For most systems, pathological complete response (pCR) describes an absence of any signs of cancer on histological resection specimen. Good (GR) and bad (BR) response grades have been individually defined within each study.

In terms of findings, an area under the receiver operating characteristic curve (AUC-ROC) or c-statistic (measures of predictive performance) is provided in the findings, where possible (Table A1, Table A2, Table A3, Table A4, Table A5, Table A6 and Table A7).

## 4. Discussion

The literature for response prediction in NAT is characterised by two main approaches: the first involves using imaging techniques to compare derived variables at time points before, during and after NAT; the second approach relies on detecting molecular markers prior to NAT. Both approaches seek to influence management either through early cessation or avoidance of NAT with each group in recent years, incorporating novel techniques and modalities to identify markers of response.

### 4.1. Imaging Markers

In the past two decades, multiple systematic reviews have been published investigating the predictive potential of computed tomography (CT) and fluorodeoxyglucose-positron emission tomography (FDG-PET) [16,17]. Response prediction with CT predominantly involves comparing changes in tumour volume during NAT, whereas in FDG-PET, changes in standardized uptake value (SUV) of FDG reflect alterations in tumour metabolism. These modalities have been deemed insufficiently accurate in identifying complete pathological responders and are not recommended in clinical practice.

#### 4.1.1. Computed Tomography

Although novel CT techniques, such as 3D-CT volumetry, appear more sensitive in response prediction, van Heijl notes in a 2011 study that tumour volume fluctuates paradoxically during the course of NAT and hence should not be relied upon to accurately reflect pathological response [18]. This insight may also be key to understanding the failure of CT findings overall as a predictor of response. CT radiomics-based risk factor models have previously been explored to predict survival outcomes; however, they have demonstrated limited prognostic power over standard clinical variables [19]. This review did not identify any studies assessing the utility of CT radiomics in predicting NAT response.

#### 4.1.2. Fluorodeoxyglucose-Positron Emission Tomography

Although the OAC-specific FDG-PET studies included within this review demonstrate some efficacy in the NAT response prediction [20,21,22,23,24], it was neither accurate nor sensitive enough for clinical utility. This is consistent with conclusions drawn in recent systematic reviews that more broadly focus on oesophageal cancer [16]. Similar to CT radiomics, PET radiomic markers, such as intratumoural uptake heterogeneity, have also been explored. A study in 2013 by Tan et al. demonstrated the predictive potential of PET tumour heterogeneity markers in a low cohort study [25]. However, in a separate study by van Rossum et al. in 2016 with a larger cohort, PET heterogeneity only showed incremental predictive advantage over standard clinical prediction models [26]. Importantly, the past decade has observed a trend toward multimodal imaging techniques, such as FDG-PET-CT, and even more recently, the inclusion of magnetic resonance imaging (MRI).

#### 4.1.3. Magnetic Resonance Imaging

Recently, MRI techniques, such as diffusion-weighted imaging (DWI) and dynamic contrast enhanced (DCE) MRI have been the focus of investigation. DWI-MRI exploits the random motion of water molecules to deduce tissue cellularity and assigns the degree of diffusion with an apparent diffusion coefficient (ADC) value. Tumours that have high cellularity appear hyperintense on imaging and can be monitored through changes in ADC. DCE-MRI uses a rapid series of contrast-enhanced T1 images to track changes in tumour microvasculature (measured by area-under-concentration-time (AUC) curve for inflowing contrast).

Two studies investigating the utility of DWI-MRI in predicting NAT response concluded that changes in ADC were highly predictive of histopathological response with high specificity [27,28]. An earlier study by Kwee et al. was unable to demonstrate this but may have been limited by its low sample size. The same study did, however, demonstrate robust interobserver reproducibility with DWI-MRI results at their centre, which is encouraging and necessary for widespread adoption [29]. Concomitantly, a unimodal DCE-MRI study also demonstrated predictive potential [30]. These successes have paved the way for multimodal imaging techniques that hope to further bolster predictive accuracy. In 2018, Heethuis et al. published the first study that demonstrated the complementary value of DWI-MRI and DCE-MRI in NAT response prediction [31]. DWI-MRI and FDG-PET-CT have also been investigated in two multimodal studies and similarly demonstrated stronger combined predictive values than what each individually was capable of [32,33].

Small sample size is a universal limitation of most of the discussed MRI studies and though the quoted c-statistics are promising, one must be wary of potential type 2 errors when interpreting these results. Higher-powered multicentre studies with histologically unpooled analyses are required to confirm these findings for OACs. Borggreve et al. also identifies the 10–15 day window after starting NAT as the optimal moment for an interim NAT MRI scan, as it is timely enough for changes to manifest but early enough to avoid distortion by potential radiation oesophagitis [28], as initially theorised by van Rossum et al. [27]. In contrast, within Heethuis’s multimodality study of DWI and DCE-MRI [31], comparison between the pre- versus post-treatment MRI, as opposed to the pre-treatment versus during MRI, demonstrated higher predictive value. Interestingly, these results are inconsistent with other findings from the same institution [27,28]. This may be explained by the differences in sample size and the use of automatic contouring software to delineate the tumour rather than manual approaches used in the past [31]. Moreover, there is evidence that semiautomated delineation of the tumour on an ADC image was more reproducible than manual methods [29] and should be considered as the technique of choice for future studies.

Logistically, protocols to control for cardiopulmonary motion artefacts in DWI-MRI need to be published, as several studies noted this issue, which resulted in unusable ADC images and a further reduction in study sample size [27,30]. Furthermore, better predictive accuracy for DWI-MRI could be obtained in future studies if tumour heterogeneity were addressed by using voxel-wise approaches to map ADC and replace currently simplistic mean-based ADCs [34]. Multimodal imaging appears beneficial for better response prediction. The multicentre PRIDE study that is currently underway (ClinicalTrials.gov, accessed on 7 December 2021, Identifier NCT03474341) will investigate the utility of trimodal imaging for NAT response prediction and should secondarily validate many of the low powered MRI studies discussed in this review [35].

### 4.2. Epigenetic Biomarkers

Epigenetics refers to processes that manipulate chromatin structure to modify gene expression without fundamentally altering the DNA sequence. This is facilitated through three key processes: DNA methylation, histone modification and non-coding RNA gene interactions. MicroRNAs (miRNA) are a subset of non-coding RNA that regulate gene expression through transcriptional or post-transcriptional interactions [36]. MicroRNA and microRNA panels have been the more common avenue of inquiry in predictive epigenetic biomarkers, with only one study examining methylation and no studies on histone modification in OAC.

#### 4.2.1. MicroRNA

Multiple studies conducted preclinical investigations after using low sample size discovery cohorts to identify differential miRNA between NAT response groups [37,38,39,40,41]. In three separate papers, Lynam-Lennon et al. experimented in vitro to ascertain possible mechanisms of action for miRNA-31, miRNA-187 and miRNA-17-5p [39,40,41]. Low MiRNA-187 were associated with resistance to cisplatin and radiotherapy and, conversely, in vitro plasmid-induced overexpression promoted sensitivity. Mechanistically, multiple tumorigenic pathways, such as tumour suppressor genes and immune signalling, were implicated [39]. MiRNA-17-5p functionally modulated in vitro radioresistance and was low in patients who responded poorly to neoadjuvant chemoradiotherapy [40]. MiRNA-31 downregulation also appeared to contribute to radioresistance, but unlike miRNA-187, overexpression did not enhance sensitivity. Low levels were hypothesised to upregulate DNA repair genes, such as PARP1, SMUG1, MLH1 and MMS19 in response to radiotherapy-induced DNA damage, possibly contributing to radioresistance [41]. Bibby et al. found that miRNA-330-5p was also downregulated in non-responders and may modulate pathways that inhibit pro-apoptotic proteins [38]. Two retrospective studies have also been conducted, adding to the growing list of novel miRNAs—miRNA-192, miRNA-194 and miR-4521/miR-340-5p ratio [42,43].

Although many novel miRNA have been found, the majority of these appear to be from the same discovery cohort used in Lynam-Lennon et al. and Bibby et al. and have not been clinically validated [38,39,40,41]. Moreover, Bibby et al. provides insight on the limitations of assessing miRNA’s effects on chemo- or radiosensitivity in vitro, as the NAT response in vivo involves complex interplays between the tumour microenvironment [38]. This key implication further emphasizes the importance of validating novel miRNA’s in larger cohorts to ensure generalisability before commencing preclinical studies to elucidate mechanisms. Given the similar discovery cohorts used between a large portion of miRNA studies and the lack of validation [38,39,40,41], there is inconclusive evidence to suggest any promising singular miRNA biomarkers for NAT response prediction in OAC.

#### 4.2.2. MiRNA Panels

Despite the discovery of many individual miRNA, to date there are only two OAC-specific miRNA panels that have been evaluated [44,45]. In 2015, Odenthal et al. generated a panel from a discovery cohort, which included previously investigated miRNA-192. Unfortunately, the panel failed to demonstrate any predictive impact [45]. The divergence in miRNA-192′s predictive potential between Odenthal’s two studies [43,45] may be attributed to several reasons. Although miRNA-192′s significance as a marker was initially validated, it was not specific to OAC, as the validation cohort consisted of 52.5% OAC patients. Odenthal’s subsequent study included miRNA-192 in a miRNA panel that failed to predict response in a cohort of only OAC patients. Furthermore, there was heterogeneity in chemotherapy regimens and serological miRNA samples were obtained in contrast to the biopsy tissue miRNA used in the first study. Despite being easier to obtain, it is unclear whether serological miRNA is reflective of tissue miRNA, as other studies have identified confounding sources of miRNA in blood [42]. Therefore, in future studies, it is important to minimise these sources of heterogeneity and focus on demonstrating predictive value of the panel before assessing whether the corresponding serological test is valid.

More successfully, Skinner et al. generated an internally validated novel miRNA expression profile (MEP) score from a panel of miRNA derived from a rigorous study design with a homogenous patient cohort to collectively determine the probability of pCR (MEP score ROC-AUC was 0.78 in model cohort) [44]. The MEP score demonstrated a novel approach to evaluating the predictive potential of combined miRNA profiles. These early successes justify further investigation into the utility of the MEP score as a predictor of NAT response [44]. Skinner et al. notes that only a partial complement of miRNA was investigated, leaving room for future studies to bolster the panel’s predictive potential by adding new miRNA.

#### 4.2.3. DNA Methylation

A reasonably powered study from Slotta-Huspenina et al. was the only study in this review to investigate DNA methylation related to NAT response in OAC and found that patients without histological response had significantly higher mean TFAP2E gene methylation [46]. Given the distinct mutational profile of OAC compared to OSCC [47], further studies should be done to investigate the difference in methylation that could explain the divergent responses to chemo- and radiotherapy between the two histological subtypes [48].

### 4.3. Genetic Biomarkers

Genetic biomarker studies have broadly been investigated in panels or as individual genes or alleles. Genes of interest are either derived from past literature or through applying a range of genetic detection techniques to discovery cohorts.

#### 4.3.1. Genes

Two retrospective studies investigated key genes to predict NAT response in an OAC specific cohort [49,50]. In one study, overexpression of CCL28 (inflammatory chemokine) and underexpression of DKK3 (a tumour suppressor and prognostic marker [51]) were predictive of pCR [49]. CCL28′s predictive value was exclusively investigated in OAC for the first time in this study and requires further validation. A key limitation of manual gene selection is that it oversimplifies complex biological processes and prevents the observation of concomitant gene interactions that may improve predictive potential.

Ephrin B3 receptor gene was another candidate biomarker identified through gene expression analysis, which enables broader oncogenic trends to be identified in functionally similar genes [50]. However, these results are limited by a lack of external validation, and the use of a lower response discrimination threshold in this study may potentially lead to false positive genes being implicated.

It is also important to note for future studies that prognostic value does not necessarily correspond to predictive potential. It is difficult to differentiate between these outcomes without a surgery-only treatment arm in prospective studies, which is becoming less possible as NAT has shown clear benefits in increasing overall survival [5].

#### 4.3.2. Gene Panel

A panel of 26 differentially expressed genes was used to create an artificial neuronal network (ANN) with predictive potential in a recent pilot study by Lloyd et al. [52]. ANN has previously been used in one study to investigate differential genes; however, these genes were manually selected, and the study did not focus on OAC [53]. Lloyd et al. demonstrates a novel approach to predicting NAT response in OAC with an ANN that preliminarily evaluated a 26-gene panel with an accuracy of 73%, sensitivity of 80% and specificity of 70% [52]. Further studies in larger cohorts are recommended to validate the ANN model and improve predictive performance.

#### 4.3.3. Alleles

The alleles and single nucleotide polymorphisms (SNPs) investigated in the literature are largely heterogeneous and based on past evidence of predictive potential in other cancers [54,55,56]. Although sample sizes were large relative to other biomarker studies, most articles had mixed histology, albeit more OAC than OSCC [55,56,57].

Heterozygous ERCC1, which codes a component of a nucleotide excision repair complex that repairs platinum-induced DNA damage [54], demonstrated positive association in an OAC specific study but could not be confirmed in a recent prospective study due to incompletely reported results [57]. Hence its predictive performance remains statistically unquantified and should be confirmed in a future prospective study.

To add to the heterogeneity, one study found significantly reduced NAT response with vitamin D receptor polymorphism (Apal) [56], while another found no significance with the multidrug resistance protein gene, ABCB1 [55]. These findings were not corroborated by any other study identified within this review and have not been externally validated.

Furthermore, as 5FU and cisplatin act in a p53-dependent fashion [58,59], Kandioler explored the effect of mutant TP53 on NAT response and found significant difference in response between patients with normal and mutant TP53 [60]. Clinical validation of TP53′s status as a predictive biomarker is currently being explored in the multicentre Pancho trial (ClinicalTrials.gov, accessed on 7 December 2021, Identifier: NCT00525200) [61].

#### 4.3.4. Copy Number Alterations

Qian et al. reported the first use of genomic gains as an independently validated biomarker [62]. Copy number increases in chromosome 14q11 and 19p13 were significantly associated with pCR [62]. Conceptually, copy number alterations present an interesting avenue of further genetic research, given these findings and the role of radiation-induced instability, which has been previously hypothesised as a mechanism of miRNA dysregulation [41].

### 4.4. Protein Expression

Protein biomarkers are a heterogeneous group of molecules that are categorised in this review by their potential role in oncogenesis.

#### 4.4.1. Growth and Proliferation

Epidermal growth factor receptor (EGFR) dysregulation results in increased cell survival, proliferation and migration [63]. Aichler et al. supports the hypothesis that EGFR overexpression contributes to cisplatin-based resistance [63]. However, less than 40% of non-responders exhibited overexpression, suggesting that EGFR alone is not enough to accurately predict NAT response.

The sonic hedgehog (SHH) pathway is a key growth signal for epithelial-mesodermal interactions during embryonic gut development. Past studies suggest that in both OAC and OSCC, SHH is overexpressed due to possible genomic amplification [64]. A validated immunohistochemistry-based assay for SHH pathway proteins demonstrated high AUC-ROC values in a second validation study with consistent results to the first [65,66]. This assay also used labelling scores that minimise interobserver variability. A few minor limitations exist, including the use of older 5FU-based chemotherapy regimens rather than those with less toxicity, such as CROSS [5], although the authors do not anticipate this to significantly impact the test. Regardless, this remains to be clinically validated. A prospective phase II trial (ClinicalTrials.gov, accessed on 7 December 2021, Identifier NCT04018872) will further explore the correlation of hedgehog pathway markers with therapy response by using itraconazole as a signalling pathway inhibitor.

#### 4.4.2. Metabolic Dysregulation

Changes in mitochondrial protein expression alter NAT sensitivity and affect response [67,68]. Reduced mitochondrial electron transport chain (ETC) protein expression was correlated to increased cisplatin response [68], while overexpression of ATP5B, a marker of oxidative phosphorylation, demonstrated poor pathological response and radioresistance [67].

Oxidative stress contributes to oncogenesis and cancer progression by activating a plethora of transcription factors, such as p53 and NF-kB that have been independently investigated as predictive biomarkers [66,69,70]. Alterations to ETC proteins and ATP5B may reflect the same treatment-resistant cellular response caused by oxidative stress. However, ETC underexpression has only been validated in chemotherapy-only patients, whereas ATP5B as a radioresistance biomarker has only been observed in vitro [67,68]. Contrastingly, the thioredoxin interacting protein, a redox buffer and surrogate marker of oxidative stress, yielded no significant difference between NAT response groups in Woolston et al. [71]. Although this appears to contradict the hypothesis that oxidative stress alters NAT sensitivity, their patient sample included a potentially confounding group of GOJ and gastric cancer patients [71]. Despite preliminary studies demonstrating promising predictive potential for oxidative stress markers, further validation is required in studies using newer chemoradiotherapy regimens, such as CROSS or FLOT before any conclusions can be drawn [5,10].

#### 4.4.3. Anti-Apoptosis

Two studies highlighted anti-apoptotic proteins that modulated chemo- or radio therapy response in preclinical settings [72,73]. Piro et al. demonstrated in OAC cell lines that downregulating BIRC3, a direct caspase inhibitor in apoptosis, with a transforming-growth-factor-*β* activated kinase 1 (TAK1) inhibitor increased chemo- and radiosensitivity [72]. The autophagy markers, LC3B and p62, taken together as an ‘autophagic index’, demonstrated non-response to chemotherapy if p62 was high, both alone or together with low LC3B [73]. Unfortunately, the study was limited by incomplete validation due to compromised tissue samples. Given these findings, the studies suggested TAK1 and p62 inhibitors to be investigated as potential sensitising agents in chemoradiotherapy [72,73]. Unfortunately, both studies lack external validation for BIRC3 and autophagy markers to be serious candidates for NAT response prediction. Higher BIRC3 expression in OAC compared to OSCC, however, could suggest that BIRC3 contributes to the divergence in NAT response between the two histological subtypes [47] and thus presents an avenue to elucidate molecular differences between the two cancers.

Moreover, two different studies found that SCCA1 overexpression increased chemoresistance [74,75]. Specifically, high SCCA1 levels were clustered in patients with higher TRGs [74]. SCCA1 is believed to have an anti-apoptotic affect against the lysosomal membrane permeabilisation response to cell stress [76]. In a recent externally validated preclinical study, SCCA1 has also been directly and inversely associated with peritumoural leptin and immune activation markers, respectively, as well as PD-L1 expression [75]. These findings suggest a role for SCCA1 in tumour, microenvironmental and immune cell crosstalk and may be a key marker for future studies in understanding these interactions.

Despite OAC’s genetic differences to OSCC and gastric cancer, there is mounting evidence that increased nicotinamide N-methyltransferase (NNMT) enzyme expression may play a pivotal role in cancer growth, metastasis and chemoresistance [77]. NNMT is involved with the catabolism of structurally related compounds, such as nicotinamide and pyridine, allowing urinary excretion of these products [78]. Nicotinamide has been described to have an inhibitory effect on histone deacetylases and poly(ADP-ribose) polymerases (PARPs), which promote genomic stability in response to acquired DNA damage through genotoxic events, such as radiotherapy [79]. Therefore, it has been hypothesised that NNMT overexpression and subsequent reduction of intracellular nicotinamide in cancer stem cells removes PARP inhibition, conferring improved survival characteristics of cancer stem cells against DNA damage and programmed cell death [77].

In a preclinical study on cancer stem cells, Pozzi et al. identified NNMT to be associated with cancer stem cell enrichment in a variety of epithelial and mesenchymal cancer cell lines, including bladder, lung, colorectal and osteosarcoma [77]. Moreover, further work in melanoma cell lines found that NNMT silencing through enzyme knockdown conferred chemosensitivity, highlighting it as a potential molecular target for future chemotherapeutic agents [80]. These findings were also supported in OSCC cell lines by Cui et al. who demonstrated that NNMT upregulation may additionally confer chemoresistance though promoting the Warburg effect, though the underlying mechanisms that facilitate this interaction remain unclear [81]. Lim et al. also noted NNMT overexpression in gastric cancer tissue and hypothesised that post-translational modifications could explain this differential expression [82]. Although underlying molecular mechanisms are unclear, broader trends of NNMT overexpression and its effects on treatment resistance in a variety of cancer cell lines, including gastric cancer and OSCC, warrants further investigation into NNMT’s role in facilitating treatment resistance in OAC and its potential as a biomarker to predict NAT response [82,83].

#### 4.4.4. Loss of DNA Repair and Cell Cycle Regulation

Genes previously discussed, such as ERCC1 and TP53, have also had corresponding proteins examined [69,84]. Of note, p53 expression has demonstrated no correlation with NAT response in contrast to gene studies [60,84], while borderline significance was demonstrated in van Olphen et al. [69]. A possible reason for this divergent result in Fareed et al. may be due to the study’s homogeneous treatment of p53 [84]. Recent studies suggest that significant differences exist for NAT response between groups that have normal p53 compared to a mutated or absent p53 [60,61,69]. Therefore, future biomarker studies must be wary of mutant p53. Moreover, further heterogeneity existed within chemotherapy regimens and patient histology, which could also have contributed to the discordance in results [84].

#### 4.4.5. Molecular Panels

Langer et al. manually selected a panel of proteins based on prior studies to determine predictive value. From this selection, thymidylate synthetase, the target of 5FU, and MRP-1, a multidrug-resistance protein, demonstrated significant correlation with non-response to chemotherapy. ERCC1 was also studied but contrary to Fareed et al. did not show any associations with response [84,85]. Bronson et al. similarly selected a distinct panel of prognostic markers to predict pCR but discovered that marker expression was highly heterogeneous between patients and shared no correlation with NAT [86].

Contrastingly, the histopathological threshold of response defined by both studies was quite varied, with Langer et al. defining this cut-off as <50% of residual tumour left and Bronson et al. opting for pCR [85,86]. Future studies should determine their thresholds judiciously, as there is potential for false positives in Langer et al. and, inversely, a possibility for false negative in Bronson et al. This review did not find any ‘true’ molecular panel with collective predictive value.

### 4.5. Immunologic Biomarkers

Interactions between immune cells and the tumour microenvironment are a topic of recent research interest [87]. Although specific mechanisms are not yet understood, studies on predictive immunologic biomarkers are already underway.

#### 4.5.1. Immune Cells

Neutrophil-to-lymphocyte ratio (NLR) is a novel predictive biomarker that was investigated in three recent studies [88,89,90]. The studies were unanimous in demonstrating that NLR was correlated with pCR. Two studies concentrated on pre-treatment NLR but were dichotomous with defining a threshold for significance, although they also differed in tumour subtypes, chemotherapy regimens and histopathological response thresholds [88,89]. Moreover, Al Lawati et al. only investigated NLR as a secondary outcome and was not able to provide an AUC-ROC [88]. Contrastingly, Sherry et al. measured concurrent NLR changes during chemotherapy and specifically found that a high NLR in the second week of NAT makes pCR less likely [90]. Future studies to elucidate the most optimal timings to measure NLR to maximise predictive accuracy may be indicated. Overall, NLR has thus far shown consistency in response prediction despite differences between studies, although a validation of its efficacy within an OAC-only prospective cohort treated by newer trimodal NAT regimes is indicated.

#### 4.5.2. Immune Markers

Over the last few years, several novel immunologic biomarkers have been investigated in vitro to demonstrate radioresistance [91,92,93]. Most prominently, ADAM12 is a biomarker for cancer-associated fibroblasts (CAF) found in the tumour stromal microenvironment. Ebbing et al. showed that CAF-induced IL-6 conferred chemoradioresistance to in vitro cells treated with CROSS regimen [91]. High levels of ADAM12, as well as the complement C3 and leukaemia-inducible factor (LIF) presented in other studies are all associated with poor NAT response [92,93]. Of the three biomarkers, only LIF has been internally validated but with a small cohort [92], although ADAM12 may soon be externally validated in an upcoming clinical trial (ClinicalTrial.gov, accessed on 7 December 2021, Identifier NCT04554771). However as of now, each of these preliminary studies require external validation to confirm their effects. Furthermore, these immune markers can all be derived from blood samples, making them advantageously easier to obtain and monitor compared to tissue biomarkers.

#### 4.5.3. Immune Panels

This review identified one immunogenetic signature investigated by Ghatak et al., which when combined with five differentially expressed genes in a 31 patient cohort, predicted NAT response with AUC-ROC of 0.96 [94]. Although these results are promising, they were presented in a conference abstract and therefore should be interpreted cautiously and should be verified in larger cohorts.

### 4.6. Blood and Serum Markers

Recent studies have shown interest in novel blood markers, such as circulating tumour DNA (ctDNA) and circulating tumour cells (CTCs) as well as adapting older biomarkers, such as CEA and CA19-9 to predictive uses in OAC [95,96,97]. In past studies, non-specific markers, such as plasma lipoproteins, albumin, fibrinogen and platelets have also been explored [98,99,100].

#### 4.6.1. Circulating Tumour Markers

Circulating tumour DNA is a subset of total circulating cell-free DNA and has been touted, along with CTCs to have “liquid biopsy” potential [95,101]. Although there is some evidence to support the predictive value of CTCs and ctDNA in other cancers, there are few studies that investigate their efficacy in OAC [101,102]. Egyud et al.’s results were limited by a small dataset and a low ctDNA detection rate hampered further by the unavailability of DNA from patients for verification [95]. Despite demonstrating some evidence of ctDNA correlation with NAT response, study limitations prevent any robust conclusions from being drawn. As noted with other studies where blood draws are required, future studies require protocols to ensure homogeneity for when blood is collected [90,95]. This factor further contributed to the limitations of this study.

The predictive potential of CTCs has been more extensively studied in gastric and colorectal cancers, less so in OAC. Several barriers currently exist for widespread adoption of CTC detection, specifically, faster, cheaper and more accurate CTC detection devices are needed along with the establishment of CTC detection protocols [101]. This review identified a conference abstract by Seyedin et al., who used a nanotechnology-based CTC capture system to detect a reduction of CTC levels during NAT to predict response but again, limits with sample size and a heterogeneity in tumour types prevented meaningful conclusions from being drawn [97]. An upcoming case-control study (ClinicalTrials.gov, accessed on 7 December 2021, Identifier NCT02812680) aims to further assess CTCs’ feasibility as a predictive marker. A contrastingly larger cohort in van der Kaaij et al. demonstrated that concurrently high CEA and CA19-9 levels were predictive of NAT non-response with high specificity [96]. If successfully validated in prospective studies, high CEA and CA19-9 could prompt a reconsideration of NAT in the context of other clinical findings.

#### 4.6.2. Plasma and Serum Markers

Non-specific markers, such as plasma lipoproteins, albumin, fibrinogen and platelet counts have also been investigated for predictive potential in three studies [98,99,100]. The issue with non-specific markers is that probable confounders may influence their accuracy. Kelly et al. partially accounts for this by suggesting the use of a triple plasma protein panel [98]; however, none of these studies have externally validated their findings yet.

### 4.7. Miscellaneous Markers

#### 4.7.1. Adipocytes and Leptin

Tumour leptin as a biomarker was identified initially through gene expression analysis by Bain et al., who then found that higher leptin expression demonstrated significant association with NAT non-response as well as cisplatin resistance [103]. In vitro investigations involving leptin receptor antagonists subsequently demonstrated increased cisplatin sensitivity, which suggests that they could be useful in combination with cisplatin-based chemotherapy. Additionally, visceral obesity has been reported to increase radioresistance by stimulating spindle formation during anaphase [104]. Paradoxically, obesity does not appear to be predictive of poorer disease outcomes and, in fact, may even be associated with reduced risk of acute NAT toxicities [105]. Further studies should aim to clarify these findings.

#### 4.7.2. Cancer Stem Cells

ALDH1 is a marker of cancer stem cells (CSCs), which are a chemoresistant cell population present in many cancers [106]. Ajani et al. scored each histological specimen based on ALDH1 presence to establish a labelling index which, when scored highly, suggested non-pCR [107]. This was congruent with expectations, as high ALDH-1 levels suggest high densities of chemoresistant CSCs. Given the strong predictive potential presented in this study, external validation should be pursued.

#### 4.7.3. Tumour Proportions

The relative proportion of tumour cells to intratumoural stroma demonstrated efficacy as a predictive biomarker in Hale et al. [108]. Interestingly, high proportions of tumour to stroma were associated with a poorer NAT response [108]. Unfortunately, Hale et al. included both OAC and OSCC within the study, making it difficult to ascertain differences in tumour proportion between them. Future prospective studies would be useful to confirm these findings and clarify this difference between tumour subtypes.

#### 4.7.4. Organoid Cultures

Organoid cultures are a novel in vitro technique that may facilitate preclinical studies that are better representations of NAT interactions within an in vivo tumour environment compared to conventional cell lines. The OPPOSITE study seeks to correlate in vivo to in vitro responses to NAT and elucidate potential biomarkers in the process (ClinicalTrials.gov, accessed on 7 December 2021, Identifier NCT03429816).

### 4.8. Limitations

This review was limited to articles published from 2010 onwards, as this allowed more focus to be given to studies using clinically relevant chemotherapy regimens. This may have caused a biased perspective on biomarkers that had more evidence published prior to 2010. However, there are several reviews that have a better focus on biomarkers published prior to 2010 and it is recommended that the evidence provided for older biomarkers be reviewed in conjunction with past reviews.

Secondly, although studies were critically appraised, a validated tool was not used. Instead, appraisal was guided by a 10-question appraisal process outlined by Young et al. to uphold rigor [109]. Nevertheless, objective quality comparisons between studies could not validly be made.

## 5. Conclusions

This review presents a heterogeneous landscape of predictive biomarkers that have been studied in the last decade. Although recent developments in imaging and immunologic markers have been promising, no biomarker currently has sufficient evidence to support clinical application. The shift from single biomarker studies to investigating biomarker panels or signatures will likely be driven by increasingly sensitive detection methods that will contribute dozens of differentially expressed biomarkers. Although concurrent validation of existing biomarkers needs to occur, tools, such as artificial neuronal networks, are also required to efficiently assess the predictive potential of a growing crowd of heterogeneous molecular biomarkers. To provide better clarity within the field, this review presents the following recommendations for future studies.

### 5.1. Minimise Heterogeneity Where Possible

- OAC and OSCC should be treated as different entities; future studies should either focus on a specific histological subtype or provide unpooled subtype analyses.
- Tumour locations, such as oesophageal, gastro-oesophageal and gastric should similarly be differentiated.
- TRG classification systems should aim for standardisation across the literature along with a definition of a ‘good’ and ‘bad’ response to allow for more valid comparisons between studies. To reduce the effect of interobserver variability with TRG between institutions, consider comparing Mandard’s TRG1-2 to 4-5 for detecting biomarkers.
- Consistency with NAT regimen between patients.

### 5.2. Clinical Validation

- All preclinical studies should aim to include an internal validation cohort.
- All studies are recommended to pursue external validation.

### 5.3. Future Research Directions

- Biomarker panels may provide better-combined predictive potential than singular biomarkers, just as multimodality imaging has improved predictive value of imaging.
- Development of artificial neuronal networks for multi-molecular biomarker panel assessment.
- Use of organoid cultures to identify biomarkers of interest.
- Investigation into the predictive potential of the biomarker NNMT in OAC.
- Implementation of robust predictive models that integrate data from different diagnostic streams (e.g. radiological and pathological response indicators).

## Appendix B

**Table A1:** Imaging markers.

Year	Biomarker	Tumour Type and No. of Patients	NAT Regimen	TRG (GR v BR)	Study Type	Findings	Reference
**CT**							
**2011**	3D-CT	33 OAC; 6 OSCC	CROSS	Mandard (1–2 vs. 3–5)	Prospective	The 14-day tumour volume change was not statistically significant between response groups in terms of TRG. AUC-ROC = 0.71. CT volumetry not recommended for response assessment.	Van Heijl [18]
**FDG-PET(-CT)**							
**2010**	PET *	-	MAGIC (ECX)	Mandard	Prospective	Preliminary results of FDG-PET measured metabolic response could predict histopathological response.	Bain [20]
**2011**	PET	119 OAC; 26 OSCC	CROSS	Mandard (1–2 vs. 3–5)	Prospective	AUC-ROC = 0.71 in OAC patients, very similar to the AUC-ROC overall. NPV = 75% at 0% cut-off. If 20% or 30% cut-off used, half of patients were incorrectly identified by PET to be non-responders. No association between reduced SUV and NAT response but accuracy and NPV was too low to be clinically useful.	Van Heijl [21]
**2012**	PET-CT	38 OAC; 8 OSCC; 2 Other	Oxaliplatin +5FU	Mandard (1–3 vs. 4–5)	Prospective	Subgroup analysis of OAC demonstrates PET/CT metabolic response significantly associated with histopathological pathological response. Low sensitivity (55%).	Gillies [22]
**2012**	PET-CT	66 OAC	MAGIC (ECF/ECX)	Schneider (1–2 vs. 3–4)	Retrospective	NAT responders identified at a 79% true negative, and 75% true positive rate at a >67% SUV change cut-off. True negative of 100% and 33% true positive at a >33% cut-off. FDG-PET not accurate enough to predict NAT responders. AUC-ROC = 0.810.	Kauppi [23]
**2013**	PET Radiomics	18 OAC 2 OSCC	CALBG 9781	Mandard	Retrospective	Various PET tumour features including: SUV intensity distribution, texture and geometry were extracted. Decline in mean SUV, skewness and certain texture features demonstrated AUC-ROC = 0.76+.	Tan [25]
**2014**	PET-CT *	6 OAC; 13 OSCC	-	-	Prospective (registered)	FDG-PET-CT measuring SUV change (SUV) did not significantly predict response. Tumour liver ratio % change was associated with response (*p* = 0.01).	Dash [24]
**2016**	PET Radiomics	217 OAC	Oxaliplatin/Doxetaxel + 5FU	pCR	Retrospective	FDG-PET-based intratumoral uptake heterogeneity as a biomarker provides incremental increase in response prediction (AUC-ROC = 0.72) compared to clinical prediction model (AUC-ROC = 0.67).	Van Rossum [26]
**2017**	PET-CT *	-	-	-	Preclinical	18F-FAZA PET/CT in OAC xenograft models predicted worse radiotherapy response. (Sensitivity = 92.3%, specificity = 71.4%).	Elodie [110]
**MRI studies**							
**2016**	DCE-MRI	21 OAC; 3 OSCC; 1 other	CROSS	Mandard (1–2 vs. 3–5) and pCR	Prospective	AUC changes are promising in predictive potential. AUC change during vs. pre-NAT was most predictive of GR (sensitivity = 92%, specificity = 77%, PPV = 79% and NPV = 91%, at a 22.7% threshold).	Heethuis [30]
**2014**	DWI-MRI	11 OAC; 1 OSCC	CALBG 9781	Mandard (1–2 vs. 3–5)	Prospective	No significant difference in mean ADC between response groups. Sample size may be too low to detect a significant correlation. Mean tumour ADC with manual measurement has good interobserver reproducibility with an intraclass correlation coefficient (ICC) of 0.69 (95% CI, 0.36 to 0.85; *p*= 0.001). Interobserver reproducibility by semi-automated volumetric measurement method was better with an ICC of 0.96 (95% CI, 0.91 to 0.98; *p* = 0.001).	Kwee [29]
**2015**	DWI-MRI	15 OAC; 5 OSCC	CROSS	Mandard (1–2 vs. 3–5) and pCR	Prospective	ADC compared during vs. baseline was significantly higher in pCR patients as well as GR patients—c-statistic = 0.90. Predictive of poor pathologic response at threshold of 21% (sensitivity = 82%, specificity = 100%, PPV = 100% and NPV = 80%).	Van Rossum [27]
**2020**	DWI-MRI	16 OAC; 8 OSCC	CROSS	Mandard (pCR)	Prospective	DWI-MRI during the 2nd week on starting neoadjuvant chemoradiotherapy is most predictive for pCR. ROC-AUC = 0.87 in second week and increased to 0.97 after several outlier patients excluded.	Borggreve [28]
**2018**	DWI-MRI and DCE-MRI	28 OAC; 4 OSCC	CROSS	Mandard (1–2 vs. 3–5) and pCR	Prospective	DWI-MRI ADC change post- vs. pre-NAT yields c-statistic of 0.75. DCE-MRI AUC change during vs. pre-NAT demonstrated a c-statistic of 0.79 for pCR. When combined, the complementary c-statistic increased to 0.89.	Heethuis [31]
**2018**	FDG-PET-CT and DWI-MRI	17 OAC; 3 OSCC	CALBG 9781	Mandard (pCR)	Prospective	Relative changes of ADC mean and 25th and 10th percentiles from baseline to interim completely discriminated pCR vs. non-pCR with c-statistic = 1. High inter-reader reliability. On FDG-PET-CT, changes in SUVmax showed no significant difference between NAT response groups but change in total lesion glycolysis (TLG) during vs. pre-NAT did with a c-statistic of 0.947.	Fang [32]
**2020**	FDG-PET and DWI-MRI	57 OAC; 11 OSCC; 1 other	CROSS, CALBG 9781	Chirieac (1–2 vs. 3–5) and pCR	Prospective	Combining ADC findings from DWI-MRI during NAT, SUV from FDG-PET and histology to discriminate pCR yields c-statistic of 0.84. (individually ADC during = 0.82, SUV mean post = 0.79).	Borggreve [33]
**2021**	DWI-MRI and DCE-MRI and FDG-PET-CT	200 patients; >130 OAC	-	-	Prospective	ClinicalTrial.gov Identifier: NCT03474341. Pride study. Recruitment phase. Aims to develop multimodal model to predict probability of pCR by combining DWI-MRI, DCE-MRI and FDG-PET-CT by comparing scans before, during and after NAT.	Borggreve [35]

Biomarker studies with asterisk (*) are conference abstracts.

**Table A2:** Epigenetic markers.

Year	Biomarker	Tumour Type and No. of Patients	NAT Regimen	TRG (GR v BR)	Study Type	Findings	Reference
**MiRNA**							
**2012**	MiRNA-31	19 OAC (discovery cohort)	Cisplatin + 5FU + 40.5 Gy	Mandard (1–2 vs. 3–5)	Preclinical (with discovery cohort).	MiRNA-31 potentially affects DNA repair genes (PARP1, SMUG1, MLH1 and MMS19). Downregulated MiRNA-31 may contribute to radioresistance; overexpression did not enhance radiosensitivity.	Lynam-Lennon [41]
**2013**	MiRNA-192 and 194	16 OAC (discovery cohort); 42 OAC; 28 SCC	Cisplatin + 5FU + 40 Gy	Cologne (1–2 vs. 3–4)	Retrospective (with discovery cohort).	Pre-NAT intra-tumoural miRNA-192 and 194 was higher in OSCC and OAC, though not statistically verified. Only miRNA-192 was linked to higher TRG in OSCC patients.	Odenthal [43]
**2015**	MiRNA-330-5p	18 OAC (discovery cohort)	Cisplatin + 5FU + RTx	Mandard (1–2 vs. 4–5)	Preclinical (with discovery cohort).	miRNA-330-mediated changes to E2F1/p-AKT pathway did not significantly alter chemosensitivity. Silencing of miR-330-5p enhanced, albeit subtly, cellular resistance to clinically relevant doses of radiation.	Bibby [38]
**2016**	MiRNA-187	18 OAC (discovery cohort)	Cisplatin + 5FU + 40 Gy	Mandard (1–2 vs. 3–5)	Preclinical (with discovery cohort).	There are 67 differentially altered miRNA identified. Low Mir-187 was found in patients with poor NAT response. In vitro, miR-187 modulates radiation and cisplatin sensitivity and alters variety of pathways, including C3 serum levels (increased in poor responders). Supports C3 increase as predictive marker.	Lynam-Lennon [39]
**2017**	MiRNA-17-5p	18 OAC (discovery cohort).	Cisplatin + 5FU + 40 Gy	Mandard (1,2 vs. 3–5)	Preclinical (with discovery cohort).	In vitro, miR-17-5p significantly sensitises radioresistant cells to radiation and promotes repression of genes with miR-17-5p binding sites. In vivo, miR-17-5p is significantly decreased with poor NAT responders. Subpopulation of cells had low miR-17-5p, high ALDH activity and increased radioresistance.	Lynam-Lennon [40]
**2018**	MiRNA ratios	31 OAC	Cisplatin + 5FU + RTx	AJCC (0 vs. 1–3)	Retrospective	Predictive performance of miRNA ratios were analysed and miR-4521/miR-340-5p found to perform best (sensitivity = 95%; specificity = 89%). miR-101-3p/miR-451a and miR-1433p/miR-451a both had a sensitivity of 91% and specificity of 89%.	Chiam [42]
**miRNA panels**							
**2014**	MiRNA panel	10 OAC; (discovery) 43 OAC; (model) 65 OAC; (validation)	CALBG 9781	pCR	Retrospective	MiRNA profile (mir-505 *, mir-99b, mir-451 and mir-145 *). Probability of pCR plot produced, which classifies patients with very high (80%) and very low (10%) probability of pCR. MiRNA expression profile score correlated to probability plot and is a validated means of determining probability of pCR. MEP score AUC-ROC = 0.78 (model cohort), 0.71 (validation cohort) and 0.72 (combined cohort). When combined with clinical variables, the MEP score ROC-AUCs increased to 0.89, 0.77 and 0.81, respectively.	Skinner [44]
**2015**	MiRNA panel	50 OAC;	EOX, FLOT + 40–55 Gy	Cologne (1–2 vs. 3–4)	Retrospective	Based on the divergent miRNA pattern, miR-21, miR-192, miR-222, miR-302c, miR-381 and miR-549 were selected for further validation. MiRNA profile differs depending on NAT response, but failed to show significance in expanded patient cohort.	Odenthal [45]
**DNA methylation**							
**2019**	TFAP2E *	60 OAC	5FU based CTx	-	Retrospective	Higher mean TFAP2E methylation in patients without histopathological response to 5-FU-based chemotherapy (34% vs. 22%, *p* < 0.0001). AUC-ROC = 0.790 at 26.85% cut-off value of methylation.	Slotta-Huspenina [46]

Biomarker studies with asterisk (*) are conference abstracts.

**Table A3:** Genetic biomarkers.

Year	Biomarker	Tumour Type and No. of Patients	NAT Regimen	TRG (GR vs. BR)	Study Type	Findings	Reference
**Genes**							
**2010**	Ephrin B3 Receptor	47 OAC	Cisplatin + 5FU + folinic acid	Becker (1–2 vs. 3)	Retrospective	The 86 differentially expressed genes involved in cell cycle regulation, gene expression, tumour suppression, signal transduction, cytoskeleton and transcription identified on microarray. Ephrin B3 receptor had strongest difference in expression rate.	Schauer [50]
**2017**	CCL28 and DKK3	29 OAC	CROSS (93%)	Nil TRG (pCR)	Retrospective	CCL28 overexpression and DKK3 underexpression discriminates pCR from non-pCR (*p* < 0.01). CCL28 was overexpressed by a factor of 2.28 in pCR specimens, while DKK3 was underexpressed by 15% compared to non-pCR. Inhibition of DKK3 may reduce chemoresistance.	McLaren [49]
**Gene Panel**							
**2019**	Gene Panel *	56 OAC	-	Mandard (1–2 vs. 4–5)	Retrospective	Pilot study—Apoptosis and cell cycling genes upregulated in responders. Cytokine signalling and immune response genes upregulated in non-responders. The 26 differentially expressed genes were used to create an artificial neuronal network to predict NAT response. Accuracy = 73%, sensitivity = 80% and specificity = 70%.	Lloyd [52]
**Alleles**							
**2011**	ABCB1 gene polymorphism C345T	146 OAC; 116 OSCC	Cisplatin + 5FU + 36 Gy	Cologne (1–2 vs. 3–4)	Retrospective	Although 3 polymorphisms (TT, CT, CC) were associated with lymph node status and metastases, it was not predictive for response of the primary tumor to NAT.	Narumiya [55]
**2012**	ERCC1-SNP	153 OAC	Cisplatin + 5FU + 36 Gy	Schneider (1–2 vs. 3–4)	Retrospective	ERCC1 polymorphism (SNP rs11615) CT was predictive of response to NAT (*p* < 0.001). Heterozygosity suggested therapy response in 66.1% of all pts with major response.	Metzger [54]
**2016**	ERCC1-SNP *	56 OAC; 29 OSCC	Cisplatin+ 5FU + 40 Gy	Schneider (1–2 vs. 3–4)	Prospective	ERCC1-SNP with mRNA ERCC1, DPYD and ERBB2 associated with minor response to chemoradiation. Homozygous ERCC1-SNP (CC, TT) had similar minor response (70% and 75%). Heterozygous ERCC1-SNP (CT) not reported.	Bollschweiler [57]
**2018**	VDR polymorphisms	36 OAC; 16 other	-	Schneider (1–2 vs. 3–4)	Retrospective	Blood and tissue samples were assessed for Vit D levels, gene expression and polymorphisms in VDR (FokI, BsmI, ApaI, TaqI), CYP24A1 and CYP27B1. Biallelic BsmI(bb) mutation and homozygous ApaI genes (AA) were associated with reduced response to NAT. Homozygous mutant ApaI gene (aa) was exclusive to responders of NAT in OAC.	Singhal [56]
**2014**	TP53	20 OAC; 16 SCC	Cisplatin + 5FU	Complete v. partial response	Retrospective	Significant difference in response to NAT based on TP53 marker status—normal vs. mutated (*p* < 0.0001). Did not specify associations between good and bad response.	Kandioler [60]
**2018**	TP53	103 OAC; 78 OSCC	Cisplatin+ 5FU	Chirieac	Prospective	5FU and cisplatin response hypothesised to be dependent on normal TP53. TP53 mutation rate (77.9%) higher than what was expected in patients with oesophageal cancer. Results of clinical validation of predictive effect have yet to be presented.	Kappel-Latif [61]
**Genomic Copy Number Alterations**							
**2019**	Genomic gains *	52 OAC	-	pCR	Retrospective	Genomic gains in chromosome 14q11 and 19p13 were significantly associated with pCR. First biomarker study with independent validation with a focus on genomic gains.	Qian [62]

Biomarker studies with asterisk (*) are conference abstracts.

**Table A4:** Protein expression.

Year	Biomarker	Tumour Type and No. of Patients	NAT Regimen	TRG (GR vs. BR)	Study Type	Findings	Reference
**Growth and Proliferation**							
**2014**	EGFR	86 OAC	Cisplatin + 5FU + folinic acid	Becker (1 vs. 2-3)	Retrospective	EGFR overexpression and copy number gains associated with resistance to cisplatin-based neoadjuvant chemotherapy (*p* < 0.0001).	Aichler [63]
**2015**	NF-kB, SHH and Gli-1	64 OAC	5FU + taxane +/− platin + 50.4 Gy	Becker (0 vs. 1–3) AND Rohatgi (0 v 1–2 vs. 3)	Retrospective	This study validates the IHC-based assay as having good predictive value in OAC. High average AUC-ROC of 0.96 and 0.85, respectively, in two independent labs. PPV between labs was 88% and 82%; NPV at both labs was 83%. Interobserver concordance was 97%.	Rosen [66]
**2022**	Hedgehog pathway components	78 OAC/OSCC/GOJ (estimate)	-	-	Prospective	ClinicalTrials.gov Identifier: NCT04018872. Phase II Clinical Trial. Trial evaluating effectiveness of itraconazole in inhibiting hedgehog and AKT signalling pathway. Hedgehog pathway markers used to determine response to therapy.	
**Metabolic Dysregulation**							
**2013**	Mitochondrial respiratory chain proteins	23 OAC; (discovery cohort) 46 OAC; (validation cohort)	Cisplatin + 5-FU	Becker (1 vs. 2-3)	Retrospective	Reduced expression of mitochondrial respiratory chain proteins (COX7A2, COX6B1, COX6C and complex I-MLRQ) lowers threshold for cell death and is associated with increased response to treatment with cisplatin.	Aichler [68]
**2013**	Thioredoxin interacting protein	27 OAC; 20 Gastric; 41 GOJ;	MAGIC or platin + 5FU	Mandard (1–3 vs. 4–5)	Prospective	No significant difference in thioredoxin interacting protein between TRG1-3 and TRG4-5 in surgery-only or neoadjuvant chemotherapy group (*p* = 0.169).	Woolston [71]
**2014**	Mitochondrial function	23 OAC;	Cisplatin + 5FU + RTx	Mandard (1–2 vs. 3–5)	Preclinical	Alterations in mitochondrial function and energy metabolism observed in vitro, such as increased oxidative phosphorylation rates and higher ATP5B. Glycolytic markers (GAPDH, PKM2) and HSP60 were all increased in the tumour epithelium vs. stromal compartment of OAC biopsies. Proliferative differences in the two tissue compartments may exist.	Lynam-Lennon [67]
**Anti-apoptosis**							
**2015**	BIRC3	32 OAC; 33 OSCC (unpooled internal validation cohort)	cisplatin + docetaxel + 5FU + 50 Gy	Mandard (1–3 vs. 4–5)	Preclinical (with internal validation).	TAK1 inhibitor suppresses BIRC3 expression, which increased chemo- and radiosensitivity in OA cell lines. BIRC3 appears to be an important mediator of resistance. In patients, median expression of BIRC3 was (*p* < 0.0001) higher in OAC than in the more sensitive OSCC. BIRC3 expression significantly discriminated NAT sensitivity patients with OAC (AUC-ROC = 0.8074).	Piro [72]
**2017**	SCCA1	90 OAC	Platin based	Mandard (1–2 vs. 5)	Retrospective	SCCA-1 confers resistance to induced apoptosis by different mechanisms. SCCA-1 and SCCA-2 expression significantly downregulated in OAC overall. SCCA expression is significantly associated to reduced NAT sensitivity. In addition, SCCA expression has greater distribution in higher TRG OACs.	Fassan [74]
**2018**	Autophagy markers (LC3B and p62)	127 OAC	paclitaxel (in vitro) 5FU + cisplatin +/− paclitaxel	Becker (1–2 vs. 3)	Preclinical	High p62 cytoplasmic expression alone, or in combination with low LC3B was associated with NAT non-response. LC3B or p62 demonstrated no independent prognostic value post-NAT. Issues with biopsy specimens prevented tissue response prediction from being conducted.	Adams [73]
**2019**	SCCA1	56 OAC		Mandard—not specified	Preclinical study (with external validation).	OE19 cells overexpressed SCCA1 200 times more and were more resistant to docetaxel treatment. SCCA1 induces PD-L1 expression in human monocytes. OE33 cells overexpressing SCCA1 were more resistant to cell death than the control OE19 cells after treatment with epirubicin, docetaxel and cisplatin. SCCA1 increased immune activation markers.	Turato [75]
**Loss of DNA repair, cell cycle regulation**							
**2010**	ERCC1	88 OAC; 13 OSCC; 2 other	MAGIC or cisplatin + 5FU	Mandard (1–3 vs. 4–5)	Retrospective	ERCC1-positive tumours were associated with poor histopathological response to chemotherapy (*p* = 0.006). Nuclear expression of p53 was also explored—no correlation with TRG response (*p* = 0.706).	Fareed [84]
**2017**	p53, SOX2 and CD44 proteins	77 OAC (primary cohort) 70 (validation cohort)	CROSS	Mandard (1–2 vs. 3–4)	Retrospective	Aberrant p53 and SOX2 combined were significantly associated with response to NAT. Aberrant p53 expression by itself demonstrated borderline significance for predicting therapy response. CD44 expression demonstrated no significant association with NAT response in primary cohort. Primary cohort—combined markers: sensitivity = 64%, specificity = 75%, PPV = 74% and NPV = 64%.	Van Olphen [69]
**2018**	Axl *	-	-	-	Preclinical (without validation)	CKD9 inhibitor increases radiosensitivity of cells to prolonged DNA damage in vitro by enhancing G2/M arrest and apoptosis. Axl found as candidate biomarker for CDK-9 inhibition—Axl mRNA, and protein significantly reduced (52%) with CDK-9 use with radiation (*p* < 0.006).	Veeranki [111]
**Molecular Panel**							
**2010**	TS, MRP-1, ERCC1 and P-gp	40 OAC	5-FU + cisplatin +/− paclitaxel	Becker (1–2 vs. 3)	Retrospective	High TS or MRP-1 protein expression was correlated to tumour non-response to NAT (*p* = 0.001 and *p* = 0.036, respectively). For ERCC-1 and P-gp, no association between pretherapeutic protein expression and response was found.	Langer [85]
**2015**	Panel	53 OAC	-	pCR	Retrospective	Molecular biomarker panel (NF-kB, TGF-B, COX-2, Her-2/neu, p53, B-catenin, E-cadherin and MMP-1) was highly heterogeneous between pCR patients with no correlation to NAT response.	Bronson [86]

Biomarker studies with asterisk (*) are conference abstracts.

**Table A5:** Immunologic Markers.

Year	Biomarker	Tumour Type and No. of Patients	NAT Regimen	TRG (GR vs. BR)	Study Type	Findings	Reference
**Immune Cells**							
**2020**	NLR	215 OAC and GOJ	Cisplatin + 5FU + docetaxel	pCR	Retrospective	Secondary outcome demonstrated that mean baseline NLR was significantly lower in patients who had pCR (*p* = 0.009). If NLR < 1.9, likely to have pCR.	Al Lawati [88]
**2020**	NLR	136 OAC	Cisplatin + 5FU, minority—MAGIC	Mandard (1–2 vs. 3–5)	Retrospective	Pre-treatment NLR was significantly associated with a pathological response. A total of 73.5% of patients in this study had raised NLR (>2.25) and were almost 6x more likely to have a poor response to NAT. NLR had c-statistic = 0.71.	Powell [89]
**2020**	NLR	77 OAC; 16 OSCC;	Platin + taxane or 5FU + 50.4 Gy	pCR	Retrospective	NLR changes with concurrent chemotherapy are associated with response to treatment. High NLR in week 2 of NAT makes pCR less likely (OR: 0.65; *p* = 0.0076). Increasing time-dependent NLR was significantly associated with non-pCR.	Sherry [90]
**Immune Markers**							
**2018**	LIF	26 OAC	CROSS; MAGIC	Mandard (1–2 vs. 3–5)	Preclinical (with validation)	LIF was significantly elevated in in vitro radioresistant OAC cells (*p* = 0.007). Circulating LIF in pre-treatment patients was high in poor responders (*p* = 0.037), LIF mRNA expression in tumour biopsies was not significant between response groups. Radiation increased LIF secretion in vitro.	Buckley [92]
**2019**	Complement C3 *	13 OAC	-	Mandard	Retrospective and preclinical study	C3 is expressed in OAC and is significantly increased in pre-treatment OAC biopsies that have poor response to NAT (*p* < 0.05). In vitro, radioresistant cells have increased C3 mRNA (*p* < 0.01)	Cannon [93]
**2019**	ADAM12	86 OAC	CROSS	Mandard (1–2 vs. 3–4)	Preclinical	CAF induces Il-6 secretion and drives epidermal-to-mesothelial transition in vitro which confers chemoradioresistance and increased migratory capacity. Il-6 inhibition resensitised cells to therapy. Since Il-6 is non-specific, ADAM12 was found in an 86 patient cohort as a more specific marker of stromal CAFs. High serum ADAM12 was correlated with poor response to NAT (CROSS).	Ebbing [91]
**2020**	HER2, Grb7	40 HER2+ OAC	CROSS + trastuzumab + pertuzumab	-	Prospective	TRAP Phase II Feasibility Study—Grb7 positive patients demonstrated significantly better treatment response and is potentially predictive of response.	Stroes [112]
**2024**	PD-L1	56 OAC (estimate)	CROSS + durvalumab +/− tremelimumab	-	Prospective	Clinicaltrials.gov Identifier: NCT04159974. Phase II clinical trial—recruitment phase. Trial evaluating safety and efficacy of standard neoadjuvant chemoradiotherapy with immunotherapy and evaluating predictive biomarkers for response to immune checkpoint inhibition.	
**2024**	ADAM12	48 OAC (estimate)	CROSS + tocilizumab	Mandard	Prospective	ClinicalTrials.gov Identifier: NCT04554771. Phase II clinical trial—recruitment phase. ADAM12, a marker of stromal activation, will be used to assess whether stroma-targeting therapy (tocilizumab) increases efficacy of chemoradiotherapy.	
**Immune Signatures**							
**2020**	Immunogenetic signature *	31 OAC	CROSS	-	Retrospective	Identified 5 differentially mutated genes after comparing response in pre-treatment samples (EPHA5, ZNF217, RELN, PALB2 and MYO18A). Combined with 4 gene immune panel: TIM3, LAG3, IDO1 and CXCL9, which were all upregulated in responders. A risk stratification model was produced with these 9 genes to generate a c-statistic of 0.96 in NAT response prediction.	Ghatak [94]

Biomarker studies with asterisk (*) are conference abstracts.

**Table A6:** Blood and Serum Markers.

Year	Biomarker	Tumour Type and No. of Patients	NAT Regimen	TRG (GR vs. BR)	Study Type	Findings	Reference
**Circulating tumour markers**							
**2019**	CEA and CA19-9	102 OAC	Cisplatin+5FU or paclitaxel+36–50 Gy	Mandard	Retrospective	Concurrent elevation of CEA and CA19-9 was associated with early treatment failure (OR = 10.4; *p* = 0.002). Sensitivity = 0.4 (95%CI: 0.19–0.64) and specificity was 0.94 (95% CI: 0.83–0.99). PPV = 0.73 (95% CI: 0.44–0.90).	van der Kaaij [96]
**2019**	ctDNA	16 OAC	CROSS	-	Prospective	‘Proof-of-concept’ study with two patients who had pCR had baseline negative plasma ctDNA and remained disease free 500 days post-operation. Four patients selected for longitudinal plasma sequencing for ctDNA demonstrated correlation with NAT response, sometimes weeks in advance.	Egyud [95]
**2019**	CTC *	1 OAC	-	-	Prospective	Baseline CTC does not correlate with treatment response. CTCs’ reduction during NAT may predict responsive but unable to draw conclusions off sample size of 1. No comparison to pCR.	Seyedin [97]
**2021**	CTC, miRNA	200 OAC	CROSS, CALBG 9781	-	Retrospective	ClinicalTrials.gov Identifier: NCT02812680. Case-control study. Recruitment Phase. Aim: To assess the use of miRNA and CTCs as biomarkers of cancer and predictive markers for neoadjuvant therapy.	
**Plasma and Serum**							
**2010**	Plasma proteins	4 OAC; 1 OSCC	MAGIC (ECF)	-	Preclinical	Apolipoprotein A1, Serum Amyloid A and Transthyretin demonstrated significant changes (*p* < 0.05) after NAT-treated xenografts, and later confirmed in clinical samples.	Kelly [98]
**2013**	Serum albumin	211 OAC; 32 OSCC; 3 AS	MAGIC (ECF/ECX)	Mandard (1–3 vs. 4–5)	Retrospective	Malnutrition is common preoperatively, and is inversely associated with systemic inflammatory response. Hypoalbuminaemia before chemo correlates with lack of pathological response to NAT.	Noble [100]
**2015**	Fibrinogen and platelet count	56 OAC; 28 OSCC	74 CTx + 8 CRTx + 2 RTx	Mandard (1–2 vs. 3–5)	Retrospective	Significantly higher PFR (plasma fibrinogen), CRP and PBPC (peripheral blood platelet count) levels were observed in patients with good TRG. Only PFR was an independent factor influencing tumour regression.	Ilhan-Mutlu [99]

Biomarker studies with asterisk (*) are conference abstracts.

**Table A7:** Miscellaneous Markers.

Year	Biomarker	Tumour Type and No. of Patients	NAT Regimen	TRG (GR vs. BR)	Study Type	Findings	Reference
**2014**	ALDH1 labeling indices	160 OAC; 7 OSCC	5FU + Platin or taxane + 50 Gy	Chireac (pCR)	Retrospective and preclinical study	Low ALDH-1 labelling indices are predictive of pCR (*p* < 0.001; OR = 0.432), 3-fold cross-validation led to c-statistic = 0.798. High ALDH-1 has significant association with non-pCR (*p* < 0.001; OR = 3.782) and 3-fold cross-validation led to c-statistic = 0.960. In vitro studies suggest that high ALDH-1 labelling index is associated with therapy resistance.	Ajani [107]
**2014**	Tumour leptin	9 GOJ; 5 OAC; (discovery cohort) 154 OAC	MAGIC	Mandard (1–3 vs. 4–5)	Preclinical (with discovery cohort)	Gene enrichment analysis was done to identify overrepresented pathways within a cohort of 520 differentially expressed genes in radiological non-responders vs. responders. Higher leptin protein expression was associated with lack of histological response to neoadjuvant chemotherapy (*p* = 0.007). Higher leptin protein expression was associated with resistance to cisplatin (*p* = 0.008)	Bain [103]
**2016**	Adipose tissue	10 OAC	-	-	Preclinical	Anaphase bridge levels are influenced by obesity and radiosensitivity status in oesophageal adenocarcinoma. Anaphase bridges were used as a marker of genomic instability. A total of 3x more anaphase bridge in radioresistant OAC cells. Level of anaphase bridges in OE33R cells were correlated with visceral obesity status (by waist circumference and visceral fat area). Validated using spindle assemply complex genes (MAD2L2, BUB1B) in patient tumour specimens (46 viscerally obese and 41 non obese). MAD2L2 expression higher in viscerally obese.	Mongan [104]
**2016**	Tumour proportion	140 OAC and OSCC	5FU + cisplatin	Mandard (1–3 vs. 4–5)	RCT	Proportion of tumour cells per tumour area (PoT) was measured to predict chemotherapy response. PoT between 40% and 70% received survival benefit from NAT. High pre-treatment PoT related to lack of primary tumour regression (TRG4-5).	Hale [108]
**2021**	Molecular markers	40 OAC/GOJ (estimate)	CROSS	-	Prospective	ClinicalTrials.gov Identifier: NCT03429816. Organoid cultures will be used to correlate in vivo to in vitro response to neoadjuvant chemotherapy. Molecular subtypes with histological response will be correlated to identify biomarkers.	

Biomarker studies with asterisk (*) are conference abstracts.

## Appendix C

**Table A8:** Search strategy.

OVID Search Strategy		
1	((Biomarker* or Marker*) and (tumo*, biochemical, biologic*, cancer*, carcinogen*, neoplasm*, oncolog*, metabol* or predict*)).mp	1,847,563
2	((MRI or magnetic resonance imag*) and (predict* or response)).mp	281,623
3	(Neoadjuvant*, Neoadjuvant Treatment*, Neoadjuvant Therap*, Neoadjuvant Chemotherapy, Neoadjuvant Chemoradiotherapy, Preoperative Chemotherapy, Pre-operative Chemotherapy, Preoperative Chemoradiotherapy, Pre-operative Chemoradiotherapy, NAT or NAC).mp	180,001
4	(Esophageal Adenocarcinoma, oesophageal Adenocarcinoma, Adenocarcinoma of the esophagus, Adenocarcinoma of the oesophagus, Gastroesophageal Junction Adenocarcinoma, Adenocarcinoma of the Gastroesophageal Junction, GOJ Adenocarcinoma, EGJ Adenocarcinoma, Esophagogastric Junction Adenocarcinoma, oesophagogastric Junction Adenocarcinoma, esophagogastric adenocarcinoma, oesophagogastric adenocarcinoma, oesophago-gastric adenocarcinoma or esophago-gastric adenocarcinoma).mp	22,262
5	1 OR 2 AND 3 AND 4	407
**PubMed Search Strategy**		
1	(((((Biomarker*[Text Word] or Marker*)[Text Word] and (tumo*[Text Word], biochemical[Text Word], biologic*[Text Word], cancer*[Text Word], carcinogen*[Text Word], neoplasm*[Text Word], oncolog*[Text Word], metabol*[Text Word] or predict*))[Text Word])	
2	(((MRI[Text Word] OR magnetic resonance imag*)[Text Word] AND (predict*[Text Word] OR response))[Text Word]))	
3	((Neoadjuvant*[Text Word], Neoadjuvant Treatment*[Text Word], Neoadjuvant Therap*[Text Word], Neoadjuvant Chemotherapy[Text Word], Neoadjuvant Chemoradiotherapy[Text Word], Preoperative Chemotherapy[Text Word], Pre-operative Chemotherapy[Text Word], Preoperative Chemoradiotherapy[Text Word], Pre-operative Chemoradiotherapy[Text Word], NAT[Text Word] or NAC)[Text Word]))	
4	((esophageal Adenocarcinoma[Text Word], oesophageal Adenocarcinoma[Text Word], Adenocarcinoma of the esophagus[Text Word], Adenocarcinoma of the oesophagus[Text Word], Gastroesophageal Junction Adenocarcinoma[Text Word], Adenocarcinoma of the Gastroesophageal Junction[Text Word], GOJ Adenocarcinoma[Text Word], EGJ Adenocarcinoma[Text Word], Esophagogastric Junction Adenocarcinoma[Text Word], oesophagogastric Junction Adenocarcinoma[Text Word] OR esophagogastric adenocarcinoma[Text Word], oesophagogastric adenocarcinoma[Text Word], oesophago-gastric adenocarcinoma[Text Word] or esophago-gastric adenocarcinoma)[Text Word])	
5	1 OR 2 AND 3 AND 4	119
**ClinicalTrials.gov Search Strategy**		
	Search terms: neoadjuvant therapy, oesophageal adenocarcinoma, adenocarcinoma of the esophagus, neoadjuvant, esophageal, etc. Applied filters: recruiting, not yet recruiting, active not recruiting, completed, enrolling by invitation and suspended.	5 of 50 included
**Records Identified through Database Searching**		**531**

**Table A9:** Inclusion and exclusion criteria.

Inclusion Criteria	Exclusion Criteria
All peer-reviewed full-text articles and conference abstracts that were published from 2010 onwards; Articles where >50% of the patient sample had oesophageal adenocarcinoma requiring neoadjuvant therapy; Neoadjuvant chemotherapy with or without radiotherapy is a regimen that is clinically accepted at the date of authorship: FLOT, MAGIC, CROSS or CALBG 9781; ○ Variations within the same drug class were accepted (e.g., docetaxel and paclitaxel); Biomarker to predict neoadjuvant therapy outcome was investigated.	Articles where <50% of the patient sample had oesophageal adenocarcinoma were excluded if: ○ Oesophageal adenocarcinoma and squamous cell carcinoma analysis was pooled; ○ Gastroesophageal and gastric cancer analysis was pooled; Non-English articles; Review Articles.

**Table A10:** Neoadjuvant therapy regimens.

CROSS [5]	2 mg/mL/min Carboplatin + 50 mg/m^2^ Paclitaxel + 41.4 Gy Radiotherapy
MAGIC (ECF/ECX) [6]	50 mg/m^2^ Epirubicin + 60 mg/m^2^ Cisplatin + (200 mg/m^2^ 5-Fluorouracil (5FU) OR 1250 mg/m^2^ Capecitabine)
FLOT [10]	2600 mg/m^2^ 5-Fluorouracil (5FU) + 200 mg/m^2^ Leucovorin + 85 mg/m^2^ Oxaliplatin + 50 mg/m^2^ Docetaxel
CALBG 9781 [11]	100 mg/m^2^ Cisplatin + 1000 mg/m^2^/d 5-Fluorouracil (5FU) + 50.4 Gy Radiotherapy

**Table A11:** NTumour regression grade classification systems.

Mandard [9]	Becker [12]	Schneider [15]	Chireac [14]	Cologne [13]
1. Complete regression.	1a. No residual tumour/tumour bed + chemotherapy effect.	1. <1% Residual tumour cells without LN involvement.	1. No residual tumour.	1. >50% vital residual tumour cells (VRTC).
2. Sparse residual tumour cells + fibrosis.	1b. <10% Residual tumour/tumour bed + chemotherapy effect.	2. <1% Residual tumour cells with LN involvement.	2. <50% residual tumour cells.	2. 10–50% VRTC.
3. More residual tumour cells but still more fibrosis.	2. 10–50% Residual tumour/tumour bed + chemotherapy effect.	3. >1% Residual tumour cells without LN involvement.	3. >50% residual tumour cells, no response.	3. near complete regression with <10% VRTC.
4. More residual tumour cells than fibrosis.	3. >50% Residual tumour/tumour bed + chemotherapy effect.	4. >1% Residual tumour cells with LN involvement.		4. Complete regression.
5. No regression signs				
